# The ubiquilin gene family: evolutionary patterns and functional insights

**DOI:** 10.1186/1471-2148-14-63

**Published:** 2014-03-28

**Authors:** Ignacio Marín

**Affiliations:** 1 Instituto de Biomedicina de Valencia, Consejo Superior de Investigaciones Científicas (IBV-CSIC), Valencia, Spain

**Keywords:** Ubiquitination, Spermiogenesis, Ubiquitin receptors, Gene duplication, Neurodegeneration

## Abstract

**Background:**

Ubiquilins are proteins that function as ubiquitin receptors in eukaryotes. Mutations in two ubiquilin-encoding genes have been linked to the genesis of neurodegenerative diseases. However, ubiquilin functions are still poorly understood.

**Results:**

In this study, evolutionary and functional data are combined to determine the origin and diversification of the ubiquilin gene family and to characterize novel potential roles of ubiquilins in mammalian species, including humans. The analysis of more than six hundred sequences allowed characterizing ubiquilin diversity in all the main eukaryotic groups. Many organisms (e. g. fungi, many animals) have single ubiquilin genes, but duplications in animal, plant, alveolate and excavate species are described. Seven different ubiquilins have been detected in vertebrates. Two of them, here called *UBQLN5* and *UBQLN6*, had not been hitherto described. Significantly, marsupial and eutherian mammals have the most complex ubiquilin gene families, composed of up to 6 genes. This exceptional mammalian-specific expansion is the result of the recent emergence of four new genes, three of them (*UBQLN3*, *UBQLN5* and *UBQLNL*) with precise testis-specific expression patterns that indicate roles in the postmeiotic stages of spermatogenesis. A gene with related features has independently arisen in species of the *Drosophila* genus. Positive selection acting on some mammalian ubiquilins has been detected.

**Conclusions:**

The ubiquilin gene family is highly conserved in eukaryotes. The infrequent lineage-specific amplifications observed may be linked to the emergence of novel functions in particular tissues.

## Background

Mutations in the *Saccharomyces cerevisiae* gene *DSK2* were found to be suppressors of temperature-sensitive mutations in *KAR1*, a gene involved in duplication of the yeast spindle pole body [1]. Soon it became clear that *DSK2* was a member of a family of evolutionary conserved genes, present not only in yeasts but also in animals and plants, whose protein products are characterized by having a N-terminal ubiquitin-like (UBL) domain, an C-terminal ubiquitin-associated (UBA) domain and a variable number of internal Sti1 repeats [2-6] These proteins are today commonly known as ubiquilins. Mammals have several ubiquilin genes [4,7-9] Early studies demonstrated that three of them, called *UBQLN1* (formerly known also as *PLIC-1*), *UBQLN2* (a. k. a. *PLIC-2*) and *UBQLN4* (a. k. a. *A1Up*, *UBIN*, *CIP75*), are widely expressed in human, mouse or rat, while a fourth one, *UBQLN3*, is testis-specific in both human and mouse [4,6,8-12]. A fifth ubiquilin gene, called *UBQLNL*, was later detected in humans (first mentioned in [13]).

Ubiquilins are functionallly linked to the ubiquitin-proteasome system [7,14]. The UBL domain interacts with the proteasome and also with proteins containing ubiquitin-interacting motifs (UIMs), while the UBA domain serves to interact with polyubiquitinated proteins, at the same time protecting ubiquilins from proteasomal degradation [14-20]. UBL and UBA domains can also mutually interact [21]. These results suggested that ubiquilins might function as ubiquitin receptors [22], i. e. they would contact with ubiquitinated proteins either to deliver them to the proteasome for degradation or to make them enter other destruction pathways (e. g. autophagy). This has been shown to be true not only for ubiquilins, but for proteins with related structures, also containing UBL and UBA domains, such as yeast Rad23 and its ortholog proteins in other eukaryotes (reviewed in [23-26]).

Multiple results linked ubiquilins to several neurodegenerative diseases. One of them is Alzheimer’s disease (reviewed in [27,28]). UBQLN1 interacts with presenilins [6] and overexpression of either *UBQLN1* or *UBQLN2* protects presenilins from degradation [6,29]. Also, a particular polymorphism in a *UBQLN1* intron may increase the risk of suffering Alzheimer’s disease [30-37]. Additional results linking UBQLN1 with the quality control of Alzheimer’s disease-related proteins have been found recently [38-40]. Finally, reduced UBQLN1 levels were found in the brain cortex of Alzheimer’s disease patients [38]. *Drosophila* ubiquilin, encoded by the *Ubqn* (a. k. a. *dUbqln*) gene, may have related functions [41,42]. A second disease is amyotrophic lateral sclerosis (ALS). Missense mutations in the proline residues of PXX repeats present in UBQLN2 were found to cause sex-linked, dominant ALS, often associated to frontotemporal dementia [43]. Later, additional missense mutations outside those repeats were also linked to ALS [44-46]. In spinal motor neurons, UBQLN2 appears in ubiquitin-rich protein aggregates typical of ALS, both in patients with *UBQLN2* mutations and in patients lacking those mutations [43]. UBQLN1 also interacts with TDP-43, a protein involved in ALS-specific protein aggregates, and TDP-43 aggregates with either UBQLN1 or UBQLN2 in cell systems [44-47]. Similar results have been found in *Drosophila*[48]. Characteristic ubiquilin-containing aggregates are also found in ALS patients with hexanucleotide expansions in the non-coding region of the *C9orf72* gene, which is a common mutation found in both familial and sporadic ALS [49]. The fact that ubiquilins interact also with proteins involved in spinocerebellar ataxia type 1 (UBQLN4 with ataxin-1^;^[9,50]] and Huntington’s disease (UBLQN1 with huntingtin [51]), that UBLQN1 and UBQLN2 proteins are found in protein aggregates in Huntington disease models and human brains affected by Huntington and other neurodegenerative diseases [52-54] and the finding of *UBQLN1* mutations in Brown-Vialetto-Van Laere syndrome, a rare neurological disease [55], further suggested important roles of these proteins in neural tissues. However, it must be emphasized that, no matter how interesting all these results are, they probably reflect just a small fraction of the range of ubiquilin functions in humans and other mammals. For example, the broad expression patterns of *UBQLN1*, *UBQLN2* and *UBQLN4* suggest that it is likely that other tissues or organs, besides the brain, may be affected by mutations in those genes. It is also significant that the testis-specific roles of *UBLQN3* or the functions of *UBQLNL* are totally unknown. For these reasons, to determine additional roles for members of this family of proteins is a significant goal.

In this study, I analyze the patterns of diversification of ubiquilin-encoding genes in all eukaryotes, with emphasis in mammals, in which it has been found a unique expansion of this gene family. *UBQLN4* turns to be the oldest among the ubiquilin genes present in humans and other vertebrates, corresponding to the ancestral gene present in many other eukaryotes. Vertebrate species often have quite different numbers of ubiquilin-encoding genes, due to duplications and losses of these genes in different lineages. Several of these duplications, occurred in mammals, have generated a group of genes that are expressed only in testis. Functional data suggest a postmeiotic role, in spermiogenesis. These results are the first comprehensive analysis of the ubiquilin gene family available and solve the main questions regarding the origin and diversification of this family in eukaryotes. In addition, they suggest significant new views of ubiquilin functional roles.

## Results

### Global patterns of ubiquilin family evolution

Comprehensive searches, summarized in the Methods section, determined the presence in the databases of 643 full-length or almost complete ubiquilin sequences, all of them derived from eukaryotic species. So far already described in animals, plants and fungi [2,4,6], the ancient origin of this family of proteins is confirmed by the fact that they can be detected in most eukaryotic groups. They are present in both unikonts such as animals, fungi, amoebozoans, choanoflagellates or ichthyosporeans and bikonts such as plants, alveolates, stramenopiles or excavates. The presence of multiple ubiquilin genes was detected both in some plant and in some animal species. Given that organisms belonging to the sister groups of plants (green algae) and the sister group of animals (choanoflagellates) have single ubiquilin genes, it seemed likely that the expansion of the ubiquilin family in the lineages that gave rise to those plants or animals with multiple genes occurred relatively recently. This interesting question will be examined in more detail in the next Sections. A single ubiquilin gene was also found in 127 fungal species. The only fungus for which two sequences were detected was *Batrachochytrium dendrobatidis* (Chytridiomycota). However, while one of the sequences resembles the rest of fungal ubiquilins, the other one (accession number GL882891.1) encodes for a ubiquilin protein which is extremely different from the other fungal ubiquilins, moreover not resembling any other sequence in our database. Assuming it is not just a sequencing artifact, how this gene originated, i. e. whether simply emerged in a *Batrachochytrium*-specific duplication followed by drastic sequence changes or, perhaps, by horizontal transmission from another, unknown organism, is uncertain. Finally, two very different ubiquilins were detected in some excavates and in some alveolates, another result that will be described in more detail below.

An alignment of the 643 sequences was obtained (available as Additional file 1) and I performed phylogenetic analyses, either based on full-length sequences or only in the highly conserved UBL and UBA sequences. In general, the second type of analysis must be preferred when the goal is to compare very different ubiquilins. The reason is that ubiquilin sequences have a variable number of Sti1 repeats, in a way that is often unrelated to the phylogenetic proximity among species. This is an important problem if the full-length sequences are used for phylogenetic reconstruction, because the presence of exactly the same number of repeats may cause a spurious, convergent similarity among very distant sequences. However, whenever all sequences have the same structure (e. g. plants, see below), the full-length sequences can be safely used, thus increasing the amount of useful information.

**Figure 1:**
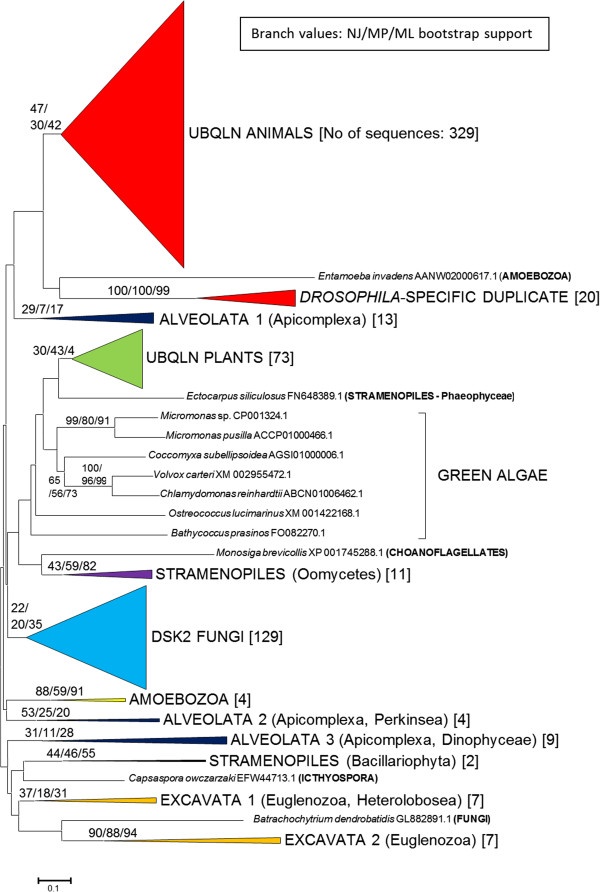
**Summary tree indicating the distribution of ubiquilins in eukaryotes.** This is the neighbor-joining (NJ) tree, but the maximum-parsimony (MP) and maximum-likelihood (ML) dendrograms were similar enough as to allow all the results to be drawn together. The tree is drawn to scale, with branch lengths in the same units as those of the evolutionary distances used to infer the phylogenetic tree. A scale bar is shown below the tree. The numbers in the branches indicate bootstrap supports (in percentages) for the three methods of phylogenetic reconstruction (as follows: NJ/MP/ML). For simplicity, only the most relevant boostrap values are indicated. In brackets, the number of sequences within each group. These groups were made by putting together all the sequences that belonged to related species, in order to deduce the minimum number of groups for each eukaryotic class. Thus, all plant can be put in a single group, animal sequences can be classified into two groups, etc. For alveolates, stramenopiles and excavates, the main phyla that can be found within each group are indicated (in parentheses). A more detailed view of this tree can be found in Additional file 2.

In Figure 1, a compact view of the trees based on the UBL and UBA domains is shown (the whole, expanded view, including species names and accession numbers can be found as Additional file 2). The results suggested that the sequences present in animals, higher plants and fungi have a monophyletic origin, given that they appear together, as three independent groups, in those trees. The only apparent exceptions are a few *Drosophila*-specific duplicates that will be discussed below and the already mentioned *Batrachochytrium* sequence. It is true that these monophyletic origins are not fully demonstrated by the analyses, given that bootstrap support for the corresponding branches is low (Figure 1, Additional file 2). However, this was not unexpected, given the limited phylogenetic signal provided by the sequences of the UBL and UBA domains, which, together, have just around 120 amino acids. In any case, alternative hypotheses, based on multiple origins, are technically possible but clearly implausible given the available data. Along the next sections, all new evidence presented is, as will become apparent, fully coherent with these hypothesized monophyletic origins.

### Diversification of ubiquilin genes in animal species

**Figure 2:**
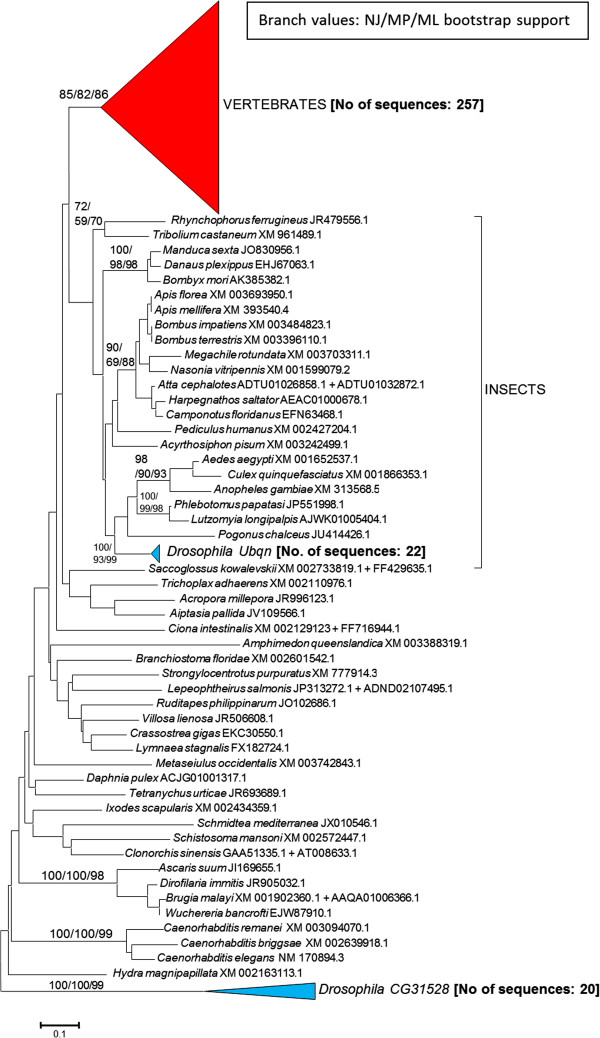
**Animal ubiquilins.** Bootstrap support (NJ/MP/ML) and number of sequences per group (in brackets) as in Figure 1. Bootstrap values are shown only for internal branches with consistent support. Scale bar as in Figure 1.

**Figure 3:**
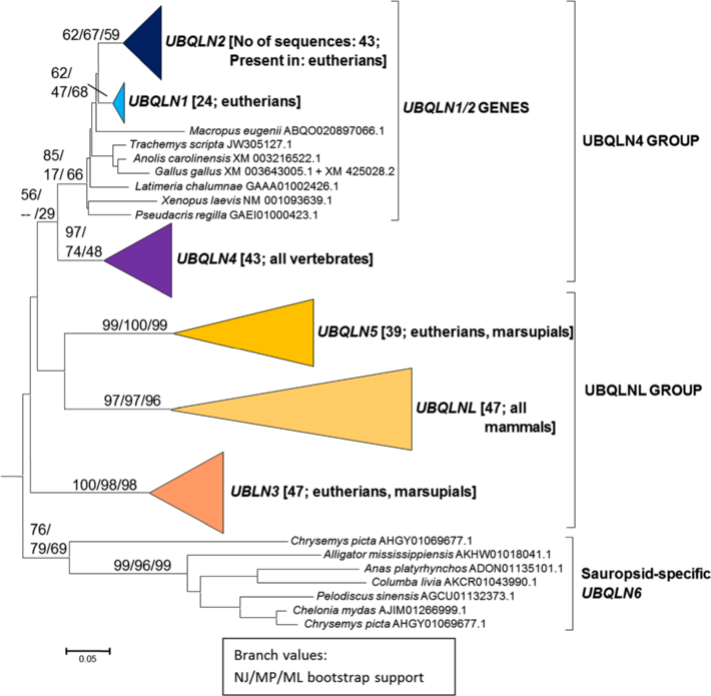
**Classification of vertebrate ubiquilins.** This figure corresponds to the subtree indicated as a triangle labelled “Vertebrates” in Figure 2, which has been expanded here, to show all the main types of ubiquilins. In brackets, the number of sequences in each group and the different types of vertebrates that have each ubiquilin gene. Bootstrap support (NJ/MP/ML) as in previous figures. Scale bar as in Figure 1.

Figures 2 and 3 summarize the results for all animal species for which ubiquilin sequences have been found. Figure 2 details phylogenetic trees, derived again from the sequences of the UBL and UBA domains, summarizing the relationships among 349 animal ubiquilins. It turned out that many animal species have single ubiquilin genes. More precisely, I found that only vertebrates and (as it was shown already in Figure 1) species of the *Drosophila* genus have two or more. As indicated above, the choanoflagellate *Monosiga brevicollis*, which is the closest animal relative among all protozoans for which data are available, also has a single ubiquilin gene. Thus, it is likely that only one gene of this family was present when animals originated.

The peculiar *Drosophila* results can be explained quite simply. One of the *Drosophila* species ubiquilin genes (called *Ubqn*) is typical, i. e. very similar to those found in all other insects (Figure 2). This gene has been the one examined in previous functional papers using *D. melanogaster* as a model [41,42,56]. The second *Drosophila* gene (named *CG31528*) clearly corresponds to a highly divergent but recently emerged, genus-specific duplicate. According to data compiled in FlyBase (http://www.flybase.org), *Ubqn* has high levels of expression in multiple tissues, while *CG31528* is highly expressed only in male testis.

A more complex situation is detected in vertebrates. Results indicate that most vertebrate sequences fit into five main classes with good bootstrap support. This is shown in Figure 3, which is simply the section of the tree shown in Figure 2 that corresponds to the vertebrate sequences, expanded. From top to bottom, the first class, indicated as “*UBQLN1/2* genes” in Figure 3, includes two human genes, *UBQLN1* and *UBQLN2*, and their orthologs, which are present in all other vertebrates except actinopterygians. The second corresponds to the set of *UBQLN4* orthologs, which appear in the tree as close relatives of the *UBQLN1/2* genes. The third one surprisingly corresponds to a hitherto undescribed ubiquilin gene. Probably, the reason for not having been detected as such before is that it is present in many mammals, but not in humans. I have called that gene *UBQLN5*. Finally, the fourth and fifth correspond to the two remnant known ubiquilin genes, *UBQLNL* and *UBQLN3*. Only a few reptilian and bird sequences are so highly divergent that do not fit well in any of those five main classes. They may correspond to a sauropsid-specific duplicate, conserved in just a handful of the species for which data are currently available (bottom of Figure 3). That kind of genes can be named *UBQLN6*.

As indicated in Figure 3, not all genes are detected in all vertebrates. On the contrary, only a single gene, corresponding to the *UBQLN4* class, was detected in all main types of vertebrates, including several actinopterygian fish species, such as *Danio rerio* or *Salmo salar*, which have this single ubiquilin gene. When we examine a closer relative of humans, the sarcopterygian *Latimeria chalumnae* (coelacanth), two genes can already be detected; one of them a typical *UBQLN4* and the second one belonging to the *UBQLN1/2* class. This situation, with two genes, is also found in amphibians, (e. g. *Xenopus*), and birds (e. g. *Gallus*)*.* In mammals, additional gene amplifications are observed, with one exception, namely the monotreme *Ornithorhynchus anatinus*. Only two *bona fide* ubiquilin genes were detected in *O. anatinus*, namely a *UBQLN1/2* gene and a typical *UBQLNL* gene. These results indicate when *UBQLNL* may have originated after the split which separated the mammalian ancestors from the rest of vertebrates. They also suggest that *UBQLN4* (which is present in species of all vertebrate groups) have been lost in *O. anatinus*. In marsupials, such as *Sarcophilus harrisii* or *Monodelphis domestica,* five ubiquilin genes (*UBQLN1/2*, *UBQLN3*, *UBQLN4*, *UBQLNL* and *UBQLN5*) were detected*.* On the other hand, most eutherians have six: *UBLQN1, UBQLN2*, *UBQLN3*, *UBQLN4*, *UBQLNL* and *UBQLN5.* Given that the presence of the two different genes, *UBQLN1* and *UBQLN2,* is restricted to this lineage, it means that they derive from a recent, eutherian-specific duplication of the precursor *UBQLN1/2* gene present in other vertebrates. Finally, as I have already indicated, *UBQLN5* -- which most likely emerged after the split that separated monotremes from the rest of mammals, given its presence in both marsupials and eutherians -- has been lost in some primates. More specifically, *UBQLN5* is found in the genomes of prosimians, such as *Otolemur garnettii* or *Microcebus murinus*. However, it has not been detected either in platyrrhines or in catarrhines, including our own species. All these results, put together, indicate that vertebrates have increased their number of ubiquilin genes from a single original one (which would correspond to *UBQLN4*) to up to 6 genes, as found today in many mammals.

Analyses of the genomic locations of these genes in multiple organisms were performed at the Ensembl web page (see Methods) and provided significant complementary information to understand their diversification in vertebrates. I first analyzed the mouse genome, finding the significant result, confirmed later in other species, that *UBQLNL*, *UBQLN3* and *UBQLN5* are located in tandem. This indicates that these three genes are evolutionary closely related, being the most likely that *UBQLN3* and *UBQLN5*, exclusive of marsupials and eutherian mammals, emerged by tandem duplications of *UBQLNL,* which is the only one detected also in monotremes. I will call from now on these three genes as the “UBQLNL group”. The second important result obtained is that the ortholog of one of the genes adjacent to *UBQLN4* in the mouse genome, called *Lamtor2*, is also adjacent to the putative *UBQLN4* gene of *Danio rerio*. This is additional evidence supporting the conclusions obtained from the phylogenetic reconstructions, indicating that all the genes that I have been hitherto calling *UBQLN4* are true orthologs and that the first *UBQLN4* gene originated before the split of actinopterygians and the rest of vertebrates. The third interesting result indicates that *UBQLN2* originated from *UBQLN1*. This derives from the study of the *Latimeria* genome. It turns out that the two coelacanth genes, which I defined above as *UBQLN4* and *UBQLN1/2* are adjacent in the genome to, respectively, *Lamtor2* (as expected, again confirming the ancient origin of *UBQLN4*) and *Idnk*. Given that this *Idnk* gene is in other mammals just adjacent to *UBQLN1* (e. g. in mouse, they are both together in chromosome 13), but not adjacent to *UBQLN2* (which is X-linked), we can conclude that the *Latimeria* gene named so far *UBQLN1/2* most likely corresponds to *UBQLN1*, with *UBQLN2* being thus an eutherian-specific duplicate. Additional confirmation is obtained from the fact that, in other species in which a single *UBQLN1/2* gene is present (e. g. *Gallus gallus*), that gene is also adjacent to the *Idnk* ortholog.

A final type of information which is relevant here concerns the protein domain structure of animal ubiquilins. As indicated in the Introduction, in addition to the terminal UBL and UBA domains, ubiquilin typically have one to a few Sti1 domains. I have explored the structures of all these proteins using the integrated tool InterProScan (see Methods). The conclusion is that UBQLN1, UBQLN2 and UBQLN4 proteins are very similar, typically having 4 Sti1 domains (although less than four are detected in some cases), while UBQLNL proteins generally have 2 Sti1 domains and UBQLN3 and UBQLN5 usually a single one, being the Sti1 domains in UBQLN3 proteins particularly divergent. Examining then invertebrate animal proteins, it was observed that almost all have 4 Sti1 domains, being thus structurally more similar to UBQLN1, UBQLN2 and UBQLN4 than to the proteins of the UBQLNL group. Given that we have deduced that *UBQLN4* is the oldest vertebrate gene, this coincidence is not surprising, and provides additional evidence supporting the monophyly of all animal ubiquilins.

**Figure 4:**
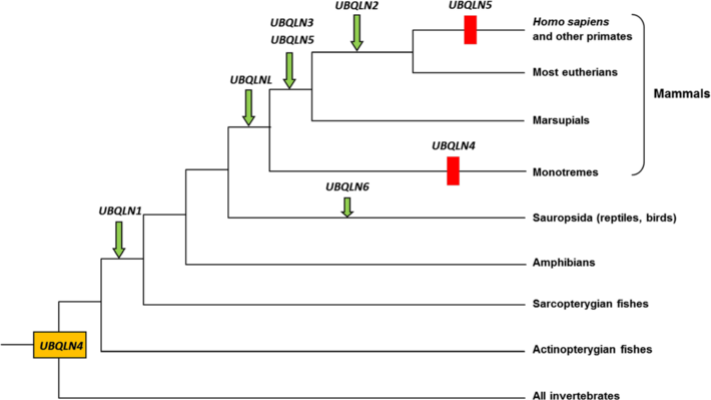
**Most parsimonious hypothesis to explain the diversification of the ubiquilin genes in animals.** Arrow: origin of a new gene. Rectangle: gene loss. A single gene, which would be orthologous to vertebrate *UBQLN4* was present when animals emerged.

Taken all these results together, it is possible to formulate the most parsimonious hypothesis that explains the whole pattern of diversification observed in vertebrates, which is detailed in Figure 4. It is important that this hypothesis agrees perfectly with all the available information (phylogenetic reconstructions, genomic evidence and protein structure results). According to those data, from a single ancient vertebrate ubiquilin gene, *UBQLN4*, which would be orthologous to the only one present in non-vertebrate animals, we deduce the early generation of a first duplicate, *UBQLN1*, followed by the origination after the mammalia/sauropsida split of four additional mammalian-specific ubiquilin genes (first *UBQLNL* and later *UBQLN2* as a *UBQLN1* duplicate, and *UBQLN3* and *UBQLN5*, which both derive from *UBQLNL*) and of the birth of a seventh gene, *UBQLN6* in sauropsids. The evidence for an exceptional mammalian-specific increase in the number of ubiquilin genes is very robust, given the already extensive data for these species groups currently present in our databases. It is interesting to point out here the fact that the three genes for which there is evidence for an involvement in human neurodegenerative diseases, either potential or direct (*UBQLN1*, *UBQLN2* and *UBQLN4*, see Introduction) have very similar UBA and UBL domains (see the small distances among them in Figure 3), encode structurally identical proteins and are related by successive duplications (UBQLN4 ➔ UBQLN1 ➔ UBQLN2). Their close relationships make advisable to call these three genes as “UBQLN4 group”. The genes of the UBQLNL group (*UBQLNL*, *UBQLN3* and *UBQLN5*) have not so far been functionally linked to any human disease.

### Evolution of ubiquilins in other organisms

In this section, I will first describe the results for the Viridiplantae, excluding the chlorophytes, which have highly divergent ubiquilins (Figure 1). The only relevant information obtained from chlorophyte sequences is that a single gene was found in seven different species and that the proteins encoded by those genes have 3 or 4 Sti1 repeats.

**Figure 5:**
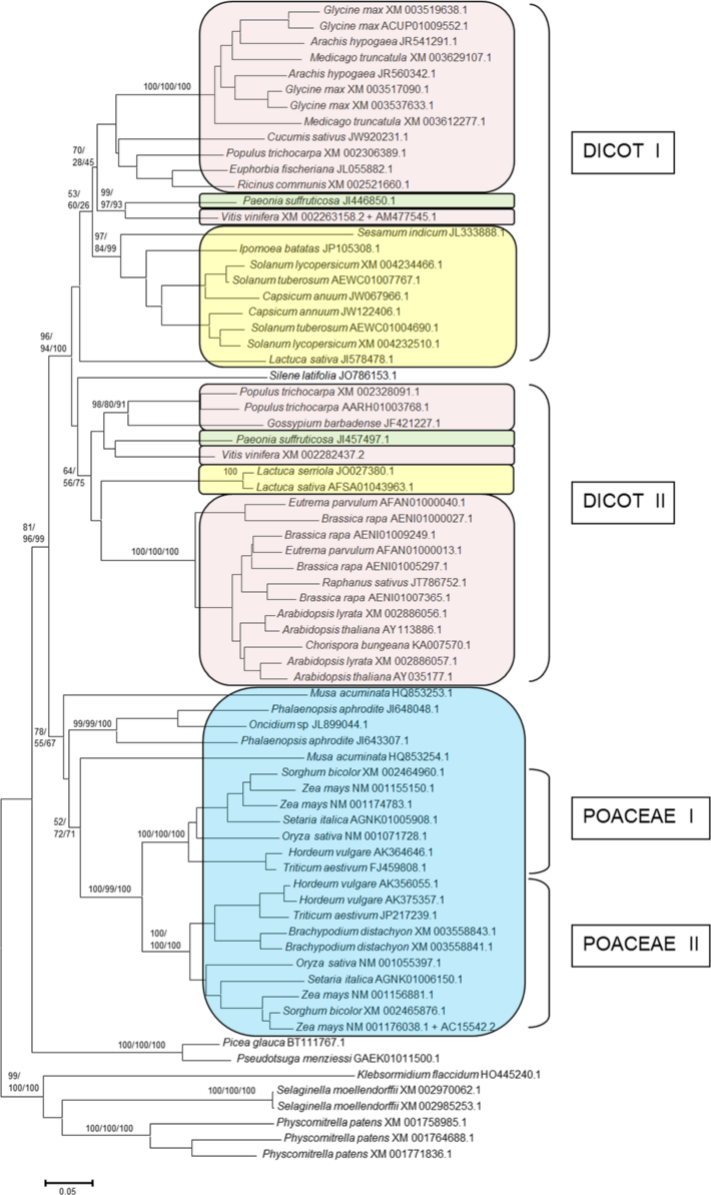
**Plant ubiquilins.** Two ancient duplications are indicated as Dicot I/II and Poaceae I/II (see text). Colors are applied to some main angiosperm classes (pink: rosid dicots; green: saxifragal dicots; yellow: asterid dicots; blue: monocots).

The number of ubiquilin sequences available in green plants is limited, just 73, but the broad phylogenetic range of species from which they derive allow for a precise characterization of their patterns of diversification. A first result is that all the species for which there is complete or almost complete genomic data have a very limited number of ubiquilins. The maximum number observed is four, in the dicots *Glycine max* and *Brassica rapa* and the monocot *Zea mays*. Most species have however just two ubiquilin genes. Figure 5 shows the phylogenetic tree obtained when those 73 sequences are compared, which serves to determine the origin of all those genes. Contrary to the trees in Figures 1, 2 and 3, which, as indicated, derive solely from the UBL and UBA domain information, this tree was obtained with the full sequences. The reason is that all plants have structurally very similar ubiquilins, all of them with four Sti1 domains. Given that they can be quite easily aligned along their whole lengths, it makes sense using here all the information to generate the trees. Notice also that this structural similarity also supports the monophyletic origin of all plant ubiquilins. This putative monophyly is again reinforced by the phylogenetic trees (Figure 5), which perfectly recapitulate the known evolutionary relationships of the plant species, with early-branching species (such as the charophyte alga *Klebsormidium*, the spikemoss *Selaginella* and the moss *Physcomytrella*) separated from both the two gymnosperms for which ubiquilin genes have been detected (*Picea glauca* and *Pseudotsuga menziessi*) and all the angiosperms. Within angiosperms, the divergence of monocot and dicot species is also recapitulated in the tree.

The simplest explanation for the results obtained is that a single ubiquilin gene was present when viridiplantae originated. After that, a few independent duplications have occurred in many lineages. Considering that the evolutionary history of many of these plants has involved multiple rounds of genome duplication, some of them ancient, it is significant that only two old duplications can be deduced from Figure 5. One of them is, which is highly supported by bootstrap analyses, is observed in the poaceae (see “Poaceae I” and “Poaceae II” in Figure 5). The second one is a duplication in dicots, produced before the splits that separated asterids (see *Lactuca* species), rosids (e. g. *Arabidopsis*) and saxifragales (see the two *Paeonia* genes). This second duplication, indicated as “Dicot I” and “Dicot II” in Figure 5, has a more limited bootstrap support, but it is the simplest way to reconcile the observed tree with the known phylogenetic relationships among all these dicot species. All the other increases in the number of ubiquilin genes that have been observed in plants can be explained by independent, very recent duplications. Notice for example the two almost identical ubiquilins found in *Selaginella* or the three similar ubiquilins found in *Physcomitrella*. The same occurs in many other species, such as *Arabidopsis thaliana*, in which there are two very similar ubiquilin genes (called *Dsk2A*/*At2g17190* and *Dsk2B*/*At2g17200*), which are located in tandem. The species with four ubiquilin genes (*Glycine max*, *Brassica rapa*, *Zea mays*) obtained that number also due to recent duplications, not observed in close relatives (as can be easily deduced from Figure 5).

Compared with the relatively complex evolutionary patterns described in vertebrate animals and in plants, the rest is much simpler, given that multiplications of the genes of this family are not detected in any other organism for which data are available. First, a single ubiquilin gene has been detected in all the remnant opisthokonts analyzed. This includes the fungal species, a choanoflagellate (*Monosiga brevicollis*) and an icthyosporean (*Capsaspora owczarzaki*). Single genes were also found in 5 amoebozoan species (from the genera *Entamoeba*, *Dictyostelium* and *Polysphondylium*). Also, a single gene has been detected in all stramenopile species characterized so far. Alveolates for which data are available have 1 or 2 ubiquilins. In species with two genes (e. g. which belong to the apicomplexan genera *Plasmodium*, *Cryptosporidium* or *Neospora*), it is clear that they are highly divergent, appearing in two distant groups in the general trees (see details in Additional file 2). This result suggests that they may derive from ancient duplications. Also, 1–2 genes are detected in excavate species, duplicates being detected in *Trypanosoma* and *Leishmania* species.

### Functional data for mammalian ubiquilins

**Figure 6:**
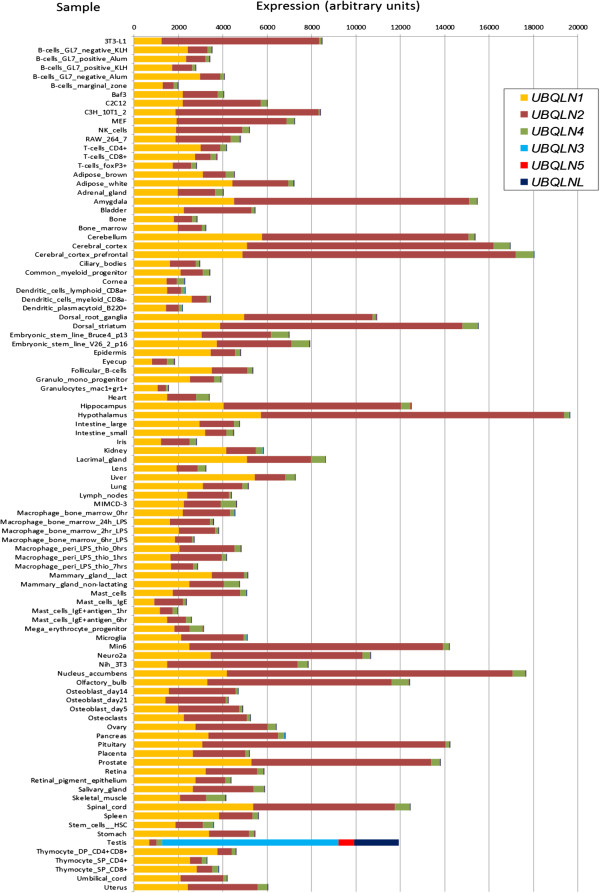
**Ubiquilin gene expression in different organs, tissues or cell types of wild-type mice.** Data, in arbitrary expression units, obtained from the BioGPS database [57]. The key names of the samples used in BioGPS are indicated. Although most names are autoexplicative, additional details of these samples can be found at http://www.biogps.org. Notice the qualitative difference found for testis respect to the other samples. In testis, most of the expression observed derives from UBQLNL group genes (*UBQLN3*, *UBQLN5*, *UBQLNL*) which are not expressed, or at very low levels, in all other tissues.

**Figure 7:**
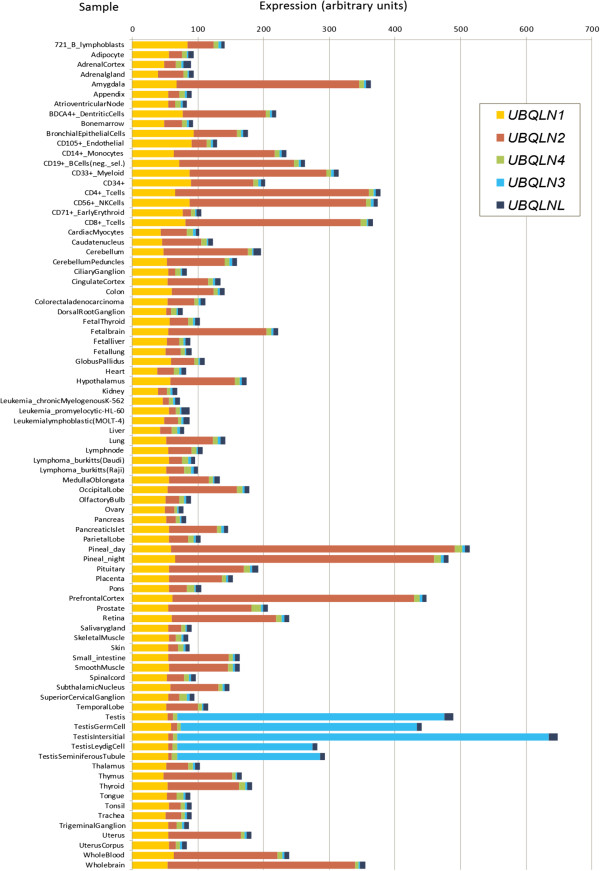
**Expression of ubiquilins in normal human tissues, organs or cell types.** Data also from BioGPS. Again, all testis-derived samples show a very high level of expression of UBQLNL group genes (*UBQLN3* and *UBQLNL*; *UBQLN5* is absent in humans).

The results in the previous two sections established that the rapid amplification of the ubiquilin gene family detected in mammals is the largest observed in any eukaryotic lineage. We may now ask for the potential roles of this novel ubiquilins that may contribute to explain such amplification. I decided to explore the available human and mouse expression data to obtain information about the potential functional roles of each ubiquilin in vertebrates. Tables 1 and Figures 6 and 7 summarize the expression data for multiple organs, tissues or cell types in, respectively, normal mice and humans. They were obtained from the last version of the Gene Atlas database available at BioGPS [57]. In Table 1 (left), I have included the details of the five mouse samples (tissues, organs or cell types) that had either the highest or the lowest levels of expression for *UBQLN1*, *UBQLN2* and *UBLQN4*. For the other three ubiquilin genes present in mouse (*UBQLN3*, *UBQLN5* and *UBQLNL*), only the five samples with the highest levels of expression are indicated, given that the values in most other samples are effectively not different from zero. The same is done for human ubiquilin genes on the right panels of Table 1. No data are indicated for *UBQLN5* given that it is absent in our species.

**Table 1:** Expression patterns of mouse and human ubiquilin genes, in arbitrary units

**MOUSE GENES**	**Expression levels**	**HUMAN GENES**	**Expression levels**
***UBQLN1*** (1424368_s_at)		***UBQLN1*** (gnf1h00141_at)	
Cerebellum	5753.64	Bronchial Epithelial Cells	93.95
Hypothalamus	5712.50	CD105+ Endothelial	90.70
Liver	5445.97	CD34+	89.30
Spinal cord	5357.54	CD56+ NK Cells	87.15
Prostate	5298.15	CD33+ Myeloid	87.05
Mast cells IgE + antigen 1hr	1166.39	Cardiac Myocytes	43.50
Granulocytes mac1 + gr1+	1052.30	Liver	42.30
Mast cells IgE	901.12	Kidney	39.60
Eyecup	809.26	Adrenal gland	38.75
Testis	691.16	Heart	38.70
Average ± s. e. m.	2651.98 ± 123.15	Average ± s. e. m.	57.58 ± 1.24
***UBQLN2*** (1450021_at)		***UBQLN2*** (215884_s_at)	
Hypothalamus	13657.53	Pineal day	431.56
Nucleus accumbens	12879.57	Pineal night	394.20
Cerebral cortex prefrontal	12305.85	Prefrontal Cortex	367.45
Min6	11411.00	CD4+ Tcells	294.45
Cerebral cortex	11112.89	Wholebrain	284.80
Thymocyte SP CD4+	538.02	Testis	8.85
B-cells marginal zone	490.66	Testis Intersitial	8.15
Cornea	450.91	Dorsal Root Ganglion	6.95
Granulocytes mac1 + gr1+	379.54	Testis Leydig Cell	6.45
Testis	318.75	Testis Seminiferous Tubule	5.95
Average ± s. e. m.	3071.72 ± 336.37	Average ± s. e. m.	78.22 ± 10.35
***UBQLN4*** (1448691_at)		***UBQLN4*** (222252_x_at)	
Skeletal muscle	854.12	Prostate	14.30
Embryonic stem line Bruce4 p13	803.26	Pineal day	11.72
Cerebral cortex prefrontal	803.07	Superior Cervical Ganglion	11.10
Embryonic stem line V26 2 p16	800.68	Pons	10.75
Olfactory bulb	776.78	Pineal night	10.58
Osteoblast day14	106.14	Kidney	5.55
Osteoblast day21	94.19	Leukemia lymphoblastic (MOLT-4)	5.50
Granulocytes mac1 + gr1+	93.57	Salivary gland	5.40
C3H 10T1 2	89.81	Wholebrain	5.15
Macrophage bone marrow 6hr LPS	69.24	Ovary	4.70
Average ± s. e. m.	309.12 ± 19.63	Average ± s. e. m.	7.39 ± 0.18
***UBQLN3*** (1436903_at)		***UBQLN3*** (220422_at)	
Testis	7940.33	Testis Intersitial	565.30
Pancreas	30.80	Testis	406.65
Cerebral cortex prefrontal	11.62	Testis Germ Cell	359.30
Salivary gland	8.30	Testis Seminiferous Tubule	216.90
MEF	6.76	Testis Leydig Cell	206.20
Average ± s. e. m.	87.92 ± 83.09	Average ± s. e. m.	23.80 ± 9.76
***UBQLN5*** (1453516_at)		***UBQLN5*** Not present in humans	
Testis	696.05		
Lacrimal gland	6.10		
Embryonic stem line Bruce4 p13	5.90		
NK cells	5.62		
Mast cells	5.49		
Average ± s. e. m.	11.93 ± 7.24		
***UBQLNL*** (1437955_at)		***UBQLNL*** (gnf1h09806_at)	
Testis	2020.45	Testis Intersitial	14.35
Granulocytes mac1 + gr1+	19.21	Testis	13.40
Cornea	10.63	Leukemia promyelocytic-HL-60	12.30
Microglia	9.19	Cerebellum	11.50
T-cells foxP3+	7.76	Adrenal Cortex	11.05
Average ± s. e. m.	26.14 ± 21.10	Average ± s. e. m.	7.56 ± 0.15

Data from the BioGPS database. In parenthesis, probes analyzed for each gene.

The results shown in Table 1 and Figure 6 complement what was known about ubiquilin expression in mouse, adding some interesting new information. First, these results confirm Northern blot expression data for mouse *UBQLN1-4*[4,8,12] which indicated that the genes of the UBQLN4 group, *UBQLN1*, *UBQLN2* and *UBQLN4,* are expressed in multiple tissues and *UBQLN3* is testis-specific. Second, it was found that *UBQLNL* and *UBLQN5* are also testis-specific (Table 1). Thus, the three UBQLNL group genes are not only evolutionarily but also functionally related. Third, it turned out that the lowest level of expression of both *UBQLN1* and *UBQLN2* among all samples analyzed actually corresponds also to the male testis (Table 1). This is not the case for *UBQLN4*: “testis” does not appear in the section of Table 1 corresponding to that gene because the level of expression in that organ (263.24) was very similar to the average level for the whole set of samples (309.12 ± 19.63; Table 1). Actually, the low standard error of the mean of *UBQLN4* values indicates a similar expression in all tissues, including testis.

Additional useful information can be obtained from Figure 6. By adding together in a single column the values of expression for all ubiquilin genes in a given tissue, some patterns become evident. Dominant in Figure 6 are the orange and brown segments, which respectively correspond to *UBQLN1* and *UBQLN2*. These two have by far the highest levels of expression among all ubiquilin genes in most tissues. The consistent but quite low expression of *UBQLN4* is, by comparison, dwarfed. It is also easily noticeable in Figure 6 how radically different from the rest of samples is the one corresponding to the testis, in which the UBQLNL group genes account for most of the expression detected. Among the genes of the UBQLN4 group, only *UBQLN1* has a relatively high level of expression in testis. Finally, it can be also appreciated in that figure that many among the samples with the highest total levels of expression, obtained adding together all ubiquilin genes, come from the nervous system (see e. g. hypothalamus, cerebral cortex, cerebellum, etc.) in good agreement with previous data [6,11].

Considering now human ubiquilins, it is important first to notice that the available information is a priori somewhat less convincing than the data for mouse genes, given that the levels of expression observed for all genes are much lower and therefore are closer to background levels (Table 1, right panels). Even with that caveat, the fact is that results very similar to those found in mouse are observed for *UBQLN2* (i. e. a broad pattern of expression, with high level in nervous system samples and low levels in testis) and *UBLQN3* (testis-specific expression, which is confirmed also by independent results [8]). The highest values for *UBQLNL* are also found in testis, although here the specificity is not as high as in mouse. Finally, some of the results for *UBQLN1* are *UBQLN4* are somewhat incongruent with those found in mouse. On one hand, *UBQLN1* is broadly expressed, but no particularly low expression in testis in detected (this is clearly seen in Figure 7). This is probably a real result, given that a relatively high level of expression in testis was observed before [11]. On the other hand, expression in whole brain samples for *UBQLN4* appears as one of the lowest. However, the values of *UBLQN4* are, as those for *UBLQNL* just mentioned, so low in all samples that is unclear to what point they are reliable. Actually, other experiments showed a relatively high level of expression of *UBLQN4* in brain [9]. In any case, even with these differences, both the obvious uniqueness of the patterns of expression observed in the testis and the fact that several nervous system samples are among the ones with the highest levels of expression, are two general results detected in both mouse and human (Figures 6 and 7).

**Figure 8:**
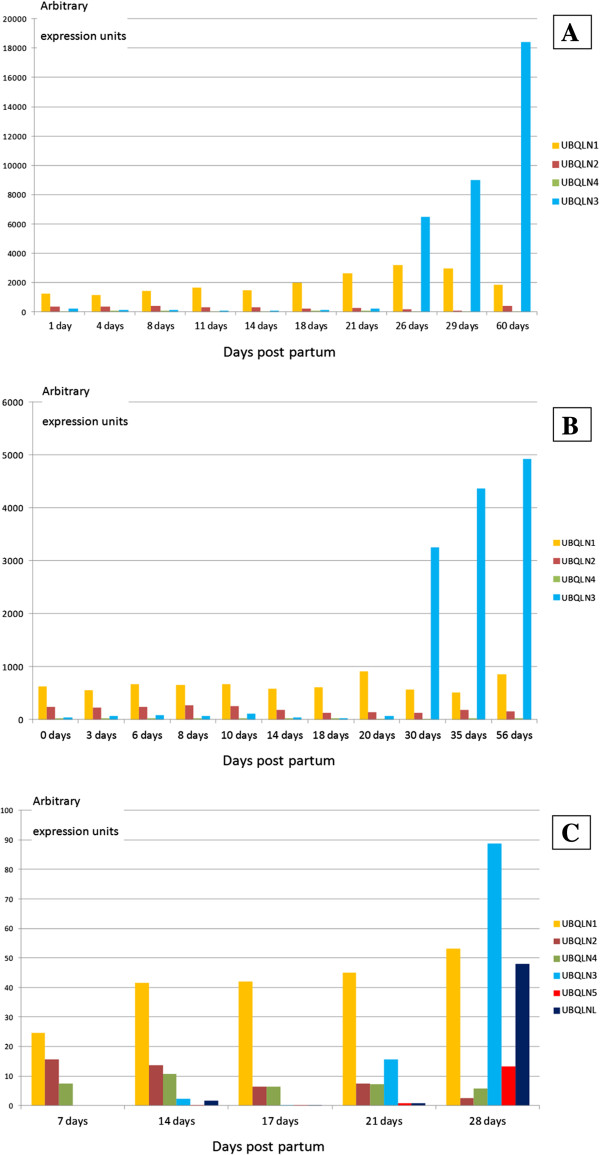
**Expression of ubiquilin genes in mouse testis, at different days post partum.** Data, in arbitrary expression units, derived from Schultz *et al.*[58]**(Panel A)**, Shima *et al.*[59]**(Panel B)** and Laiho *et al.*[60] (Panel C).

The discovery that the UBQLNL group gens are testis-specific deserves more detailed explorations. Several works have examined how gene expression changes in mouse testis after birth. Given that meiosis starts in the mouse about 10 days post partum, it is possible to indirectly assess whether testis-specific genes may be involved in pre- or postmeiotic roles by analyzing the first wave of mouse spermatogenesis, which is highly synchronic. Figure 8 summarizes results from three studies [58-60]. Although the first two are based on expression microarrays and the third one on deep transcriptome sequencing, the results coincide, and also agree well with those shown before in Figure 6. A summary is as follows: 1) *UBQLN1* has a relatively small but consistent expression in testis, from birth to adult mouse. This agrees with the data shown above in Figure 6 and also with a report indicating potential roles of *UBQLN1* in spermatogenesis [61]; 2) *UBQLN2* and *UBQLN4* are expressed at very low/null levels in testis (actually, the background levels observed are equivalent to those found for genes considered not expressed at all in that tissue [58,59]); 3) As already detected in the global results shown above (Table 1 and Figure 6), the three UBQLNL group genes are consistently expressed in the testis, with *UBQLN3* having the highest expression in the only study in which all of them were examined [60]; finally, 4) The expression of the UBQLNL group genes starts only after 20 days post partum, indicating that their products may have postmeiotic roles.

**Figure 9:**
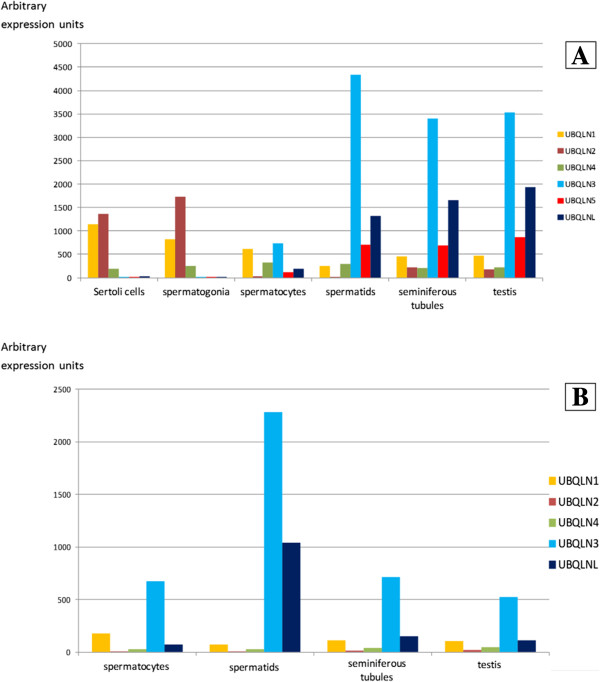
**Expression of ubiquilin genes in particular testis samples in mouse (Panel A) and human (B).** Data from Chalmel *et al.*[62], again in arbitrary expression units.

More precise assesment of those potential roles are provided by experiments devised to determine gene expression in particular cell types present in the testis. Figure 9 (top panel) shows microarray results measuring expression of ubiquilin genes in different cell types, seminiferous tubules and whole testis of the mouse [62]. In good agreement with the results presented above, expression of UBQLNL group genes is high in postmeiotic spermatids, but low or absent in spermatocytes, spermatogonia or somatic Sertoli cells. Actually, it is possible that the low level of expression detected for those genes in spermatocytes is due to contaminants, given that the authors describe the sample as “82.5% pure”. In any case, these results agree well with postmeiotic roles, in spermiogenesis, for the UBQLNL group genes. Results for human samples [62] are similar (Figure 9B). The relative lower levels in seminifeous tubules or whole testis when compared with mouse (Figure 9A) or with their own levels of expression in spermatids, may be due to an age-associated low content of postmeiotic germ line cells in the human individuals from which the samples were obtained, given that they were on average 77 years old.

### Test for positive selection acting on human ubiquilin genes

**Figure 10:**
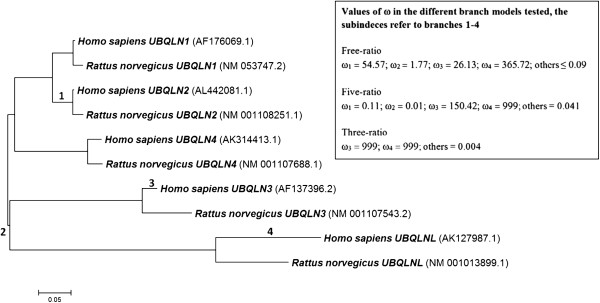
**Dendrogram corresponding to the five human ubiquilin genes and their orthologs in rat.** In parenthesis, accession numbers of the sequences. The tree shown here was obtained with the NJ method, but ML and MP analyses generated exactly the same topology. Branches 1–4 are those in which potential positive selection (ω > 1) was detected in PAML analyses. The observed ω values in the three different branch models [63] tested are also indicated. See text for details.

I checked whether positive selection was detectable on the sequences of human ubiquilin genes following standard procedures. First, the conserved UBL and UBA domains of the five human genes (*UBQLN1, UBQLN2, UBQLN3, UBQLN4* and *UBQLNL*) and their rat orthologs were aligned. The phylogenetic trees that these sequences generated were of course congruent with the expectations derived from Figure 4: *UBQLN4* may correspond to the oldest gene, while *UBQLN1* and *UBQLN2* is a relatively recent couple of paralogs and *UBQLNL* and *UBQLN3*, a second paralog duo (Figure 10). From the protein sequences of the UBL and UBA domains of those 10 genes, I obtained the corresponding nucleotide sequences and then performed codon-based analyses for positive selection using the CODEML program of the PAML package [63,64] and references therein]. Analyses were made using a recently generated graphical interface for PAML, called PAMLX [65].

Significant positive selection acting on particular codons in the whole set of sequences was not detected. Analyses implementing the six main *site models* implemented in PAML (called M0, M1a, M2a, M3, M7 and M8; see [63,64]) failed to show any result compatible with a positive selection regime. In particular, the critical comparisons [63] involving either the M1a and M2a models or the M7 and the M8 models were non-significant (not shown). On the other hand, *branch models*[63], which aim to detect positive selection acting on particular genes or gene lineages, showed more interesting results. When the simplest M0 model, which considers a single ω for the whole dataset -- being ω = d_N_/d_S_, i. e., the ratio of the nonsynonymous (d_N_) to the synonymous (d_S_) nucleotide substitution rates per codon -- was compared with a “free-ratio” model in which each branch of the tree is allowed to have a different ω value, it was found that the latter significantly improved over the first one (2 Δl = 50.53; degrees of freedom = 16; p = 0.00002; see Methods for these parameters). In the free-ratio model, ω values above 1, implying positive selection, were observed in four particular branches (labelled 1–4 in Figure 10). It is often convenient to test whether the free-ratio model, which is parameter rich, can be simplified, using models in which ω values are allowed to vary only in a few particular branches. Here, two of those simpler models were tested. The first was a “five-ratio” model in which the four potentially interesting branches detected in the free-ratio model were allowed to have their own ω values, while a fifth identical value was assigned to the rest of branches. Although this five-ratio model significantly improved over M0, the free-ratio model was still however better (2 Δl = 22.73; degrees of freedom = 12; p = 0.029). However, it was observed that only two branches (labelled 3 and 4 in Figure 4) showed values ω > 1 in the five-ratio model, which led to the idea of testing a third, “three-ratio” model, in which only three ω values were allowed, one for branch 3, another for branch 4 and a third for the rest of branches of the tree. Again, this three-ratio model significantly improved over the M0 model, but was worse than the free-ratio model, albeit with a difference that was very close to the significance level (2 Δl = 24.48; degrees of freedom = 14; p = 0.041). The ω = 999 values found in some branches in the five-ratio and three-ratio models imply a very low/zero d_S_ value. Actually, d_S_ values were 0.0001 in branch 4 of both the five-ratio and the three-ratio models and 0 in branch 3 of the three-ratio model. These very low d_S_ values however do not affect the likelihood comparisons, and therefore, the conclusion that the free-ratio model is the best one stands.

If we accept the free-ratio model as the best, this means that there is evidence for positive selection acting in four cases: 1) after the duplication that gave rise to the ancestor of the UBQLNL group genes; 2) after the *UBQLN1/UBQLN2* duplication; 3) in the *UBQLN3* genes after the rat/human split and specifically in the lineage that gave rise to humans; and, 4) in the *UBQLNL* genes, also in the human lineage. However, given that the improvement of the free-ratio model over the three-ratio model is not highly significant, it cannot be disregarded at present that positive selection may have acted solely on *UBQLN3* and *UBQLNL*, i. e. along branches 3 and 4 of Figure 10.

I finally performed *branch-site* models [63] to determine whether any codon could be detected to be under positive selection in branches 1, 2, 3 or 4 of Figure 10. Each branch was examined in an independent analysis. However, perhaps not unexpectedly, no significant results were found. The most likely reason for those negative results is that branch-site models only allow testing for positively selected codons using two different ω values, one for a given branch and a second for the rest of the tree. However, the best model observed in branch models (free-ratio model) implies a different ω value in each branch and it is thus likely that the limitation of using just two of those values for the whole tree precludes the determination of the codons under positive selection. All PAML results can be found in Additional file 3.

## Discussion

This study establishes for the first time in the literature the main patterns of the evolution of the ubiquilin gene family. All the data obtained are compatible with ubiquilins emerging very early in eukaryotic evolution and transmitting strictly vertically. The only potential exception found concerns the divergent ubiquilin detected in the chytridiomycote *Batrachochytrium dendrobatidis*, whose origin is uncertain. Very few ubiquilin genes, most likely just a single one, were present before the divergence of the main eukaryotic groups. Also, single genes were most likely present in the ancestors of animals, plants (including green algae) and fungi. Ubiquilin evolution has been in general very conservative, given that most eukaryotes have just 1 – 2 ubiquilins. The only exceptions are vertebrates and green plants, in which up to 4 – 6 ubiquilins are detected in some species. As already indicated, most plant duplications are relatively recent, often genus-specific. Just a single ancient duplication must be postulated to explain the data obtained for some monocots (poaceae) and a second one for dicots. This explains why many viridiplantae species have only 1 or 2 ubiquilin genes. Given that many plant lineages have suffered whole genome duplications, this means that ubiquilin genes are relatively “resistant” to be duplicated in plants, i. e. many of the duplicates have been lost. This is reminiscent of what is found in some families of plant ubiquitin ligases [66,67]. At present, the reason for the expansion of the ubiquilin gene family in some plant lineages is totally unknown.

Respect to all the other groups, the amplification of the ubiquilin gene family in vertebrates, and more specifically in mammals is clearly unique. Mammals have 5 – 6 ubiquilin genes, while most animal species (i. e. with the only exception of *Drosophila*, all non-vertebrates, including chordates, and also actinopterygian fish) have just one. This multiplication has occurred recently. In particular, four genes are not present in sauropsids and three of them, *UBQLN2*, *UBQLN3* and *UBQLN5*, are apparently absent in monotremes, meaning that they may have emerged in the last 150 millions of years. Losses of ubiquilin genes in vertebrates have occurred, but only rarely (Figure 4).

Genes of the UBQLN4 group (*UBQLN1*, *UBQLN2* and *UBQLN4*) have retained a general pattern of expression which must be similar to that of the single ubiquilin gene in other animals, which is most likely expressed in all tissues (e. g. the *Drosophila Ubqn* results mentioned above). There is good evidence for the products of UBQLN4 group genes having roles as ubiquitin receptors either to lead to proteasome degradation of ubiquitinated proteins [7] or to redirect ubiquitinated proteins to the autophagy pathway [68,69]. This second role is not apparently performed by the *Saccharomyces cerevisiae* single ubiquilin, DSK2 [70]. An additional facet of the role of these proteins is related to the finding that *UBQLN4* can interact with both *UBQLN1* and *UBQLN2*, and that this interaction redirects ubiquilin-interacting proteins towards the autophagy pathway [70]. This last result suggests that the roles in autophagy may depend on the presence of multiple different ubiquilins, and thus would have appeared only after the duplications that generated *UBQLN1* and *UBQLN2* from *UBQLN4*[70]. This interesting functional hypothesis could be quite simply tested, by determining whether ubiquilins have roles in autophagy in actinopterygians fishes, in which only *UBQLN4* is present. Other important points that deserve further study are: 1) why *UBQLN4* has lower levels of expression in all tissues than the other two genes (Figures 6 and 7); 2) the cause of the increased level of expression observed for these genes in particular tissues, and especially in the nervous system (see also Figures 6 and 7). This is a question that may provide strong clues about their relationship with neurodegenerative diseases; 3) whether different UBQLN4 group proteins have different affinities for different types of ubiquitinated proteins or even for particular types of ubiquitin chains, and, 4) whether there has been positive selection on the *UBQLN2* gene, as suggested by the “free-ratio” model results (see above). It is noteworthy the finding that yeast or plant ubiquilins show preferential binding to Lys-48 chains [71] while mammalian UBQLN1 binds both Lys-48 and Lys-63 chains with quite similar affinities [71,72]. Whether this difference is related to the presence in mammals of multiple related proteins of the UBQLN4 group, each with its own biochemical properties, is unknown.

The testis-specific roles of the second trio, the UBQLNL group genes *UBQLN3*, *UBQLN5* and *UBLQNL*, have been explored here in detail. A significant finding is that their main roles seem to occur in spermatids, postmeiotic germ cells (Figures 8 and 9). This may be used as a clue to understand the origin of the UBQLNL group genes, which cannot be deduced from the phylogenetic analyses, given that none of the UBQLN4 group genes is particularly similar to any of the UBQLNL group genes (Figure 3). I think that it is significant that *UBQLN1* is consistently expressed in testis (Figures 6 and 7) while the levels of expression of *UBQLN2* and *UBQLN4* are much lower. Also, it has been shown that *UBQLN1* has specific roles in postmeiotic germ cells, colocalizing with the manchette, a structure made of actin and microtubules that is present in elongating spermatids [61]. These results suggest that the oldest UBQLNL group gene, *UBQLNL*, may have originated as a duplicate of *UBQLN1.*

It is likely that the production of testis-specific tandem duplicates is linked to a strong selective pressure to increase ubiquilin gene expression in that organ. In this context, the fact that *Drosophila CG31528*, the only duplicate found in invertebrates (i. e. with an origin totally independent from that of the UBQLNL group genes) is also testis-specific seems to be more than a coincidental finding. It is also significant that some evidence for positive selection acting after the first UBQLNL group gene originated but before the *UBQLN3/UBQLNL* duplication occurred has been obtained (see “free-ratio” model results, above). The finding of positive selection acting on the *UBQLNL* and *UBQLN3* genes after the split that separated rodents from primates, specifically in the human ancestors is quite robust (Figure 10), and also deserves further study.

Two alternative hypothesis for the origin of new testis-specific ubiquilins are either that UBQLNL group genes have acquired totally novel roles in that organ (neofunctionalization) or that those genes have roles in the testis that, before their emergence, were performed by other ubiquilins, for example UBQLN1 (subfunctionalization). At present, there is no way to determine which of these options is correct, the precise roles of testis-specific ubiquilins being unknown. However, the fact that UBQLNL group proteins are structurally different from their ancestors, having lost several Sti1 domains, suggests that new functions may have arisen. In any case, this need for an increase of ubiquilins in the testis must be related to the important specific roles of the ubiquitination system in the male gonad, for which there is a growing body of evidence (see reviews: [73-75]). A recent work has pointed out that perhaps 20 % of all ubiquitin ligases, which are the proteins that provide specificity to the ubiquitination machinery, may be expressed at much higher levels or even totally specifically in the mouse testis [76]. The reasons for this particular need for a complex, testis-specific ubiquitination machinery are unknown. A hypothesis that fits well with the data obtained in this study is that this specificity may be linked to roles that are performed in male germ cells, but not in somatic cells or female germ cells. In this context, postmeiotic patterns of expression as those found here for testis-specific ubiquilins are coincidental with those of genes critical for the substitution of histones by protamines, including transition proteins and protamines themselves [77], suggesting that they might be linked, directly or indirectly, to this unique need for extreme chromatin compactation. However, other spermiogenesis-specific processes (alteration of cell shape, generation of the acrosome and flagellum, etc.; see [78]) are also candidates for involving specific roles of the ubiquitin-proteasome in which ubiquilins might be required.

Additional evolutionary considerations may contribute to discriminate among these options. It is significant that my own analyses of the data obtained by Hou *et al.*[76] indicate that those testis-specific ubiquitin ligases for which phylogenetic data is available (e. g. testis-specifically expressed members of the RBR and TRIM families that I have studied before [79,80]) originated at very different times along eukaryotic evolution (unpublished results). Therefore, the testis-specific roles of the ubiquitination system proteins that we found now in mouse or human may have arisen at very different times, making unlikely a simple, all-encompassing explanation for their functions. This means that we should look for particular, gene-specific explanations, and then it becomes relevant exactly when each particular gene originated. Thus, the fact that group UBLQNL genes are mammalian-specific, and two of them are found only in eutherians, may provide important clues about their roles. If we assume that their roles are linked to processes that only happen in eutherians, we are left with very few options, because most processes that occur in the mammalian male germ line (e. g. the histone to protamine transition indicated above) have ancient origins, being common to all vertebrates or even to all animals. Actually, the available literature indicates that the main functional differences between the male germ cells of eutherian mammals and those of reptiles, birds or even monotremes are related to the difficulty to fecundate the heavily protected eutherian oocytes (reviewed in [81,82]). Eutherian sperm has acquired a series of specific adaptations to achieve fecundation, involving redistribution of products through complex intracellular transport processes that lead to changes both in the physiology and the shape of the cells [83,84]. These specific adaptations are good candidates to require proteins with novel roles. Given that the product of *UBQLN1* – probably the ancestral gene from which the testis-specific ubiquilins emerged and also the only one among the UBQLN4 group genes consistently expressed in the testis -- colocalizes with the manchette [61], a structure linked to intracellular transport in these germ cells, an attractive hypothesis is that the emergence of the UBQLNL group proteins is related to new roles of the ubiquitination system linked to these eutherian-specific sperm adaptations. This hypothesis can be tested by generating loss-of-function mutants in the testis-specific ubiquilin genes.

## Conclusions

The ubiquilin gene family is present in all eukaryotes and has a very conservative pattern of evolution, with many eukaryotic species having a single gene. The lineage-specific amplifications observed, among which the one detected in mammals is the most extensive, are probably linked to the acquisition of specific, potentially novel functions by the newly-emerged duplicates. In mammals, three recently arisen ubiquilins are required specifically in the testis and this is also the case for a *Drosophila* novel ubiquilin gene. This suggests that duplications leading to the generation of genes with testis-specific roles may occasionally provide a selective advantage in animal lineages. Potential roles in intracellular transport for the mammalian testis-specific proteins are suggested by the available data.

## Methods

Ubiquilin sequences were obtained by TblastN searches against the nr, est, htgs, gss, wgs and tsa databases at the National Center for Biotechnology Information (NCBI; http://www.ncbi.nlm.nih.gov/). First, general searches and then specific searches to detect ubiquilins of particular groups (animal, plants, fungi, etc.) or critical model species which could have been missed in the original searches were performed. The query sequences for those searches were selected from all main eukaryotic groups in which ubiquitins were detected (animals, plants, fungi, amoebozoans, alveolates and flagellates). Given the high similarity among all ubiquilins, those searches soon became saturated, with no additional sequences being detectable when additional query sequences were used.

From those searches, and after eliminating duplicates and partial sequences, a total of 643 sequences were found to be complete or almost complete (i. e. only missing a few amino acids, generally at the N or C termini). A total of 619 of them had full-length UBA and UBL domains. These sequences were aligned using MAFFT v6.864b [85] and the alignments were manually corrected editing the sequences with GeneDoc 2.7 [86]. Phylogenetic analyses were performed following similar procedures to those in several studies focused on ubiquitination system genes ([87,88] and related references along the text). Three different methods were used, namely Neighbor-joining (NJ), maximum-parsimony (MP) and maximum-likelihood (ML). NJ and ML trees were obtained using MEGA 5.1 [89] and Maximum-parsimony (MP) trees were obtained using PAUP* 4.0, beta 10 version [90]. For NJ, Kimura´s correction was used and sites with gaps were treated with the pairwise deletion option. Parameters for MP analyses based on full-length sequences were as follows: 1) all sites included, gaps treated as unknown characters; 2) randomly generated trees used as seeds; 3) maximum number of trees saved equal to 100; and, 4) heuristic search using the tree-bisection-reconnection (TBR) algorithm, with default parameters. The same methods were used for analyses of alignments of the UBA and UBL domains, except that the faster subtree-pruning-regrafting (SPR) algorithm, also with default parameters, was used instead of the TBR algorithm. The reason for this methodological change is that the analyses based on UBA and UBL domains included a very large number of sequences, making TBR-based analyses unfeasible. Finally, for ML analyses, the BioNJ tree was taken as starting point for the iterative searches using the Jones-Taylor-Thornton (JTT) model of amino acidic substitutions. A discrete Gamma (G) distribution with five categories of sites was estimated, to account for heterogeneity in evolutionary rates. This JTT + G model was chosen because it was the best, according to the ML model comparison analyses available in MEGA 5.1. Gaps were also treated as unknown characters. Here, for the same reason indicated above, while the TBR routine with 3 levels of tree interchange was used to explore the landscape of ML trees in analyses involving full-length sequences, the faster SPR algorithm, also with 3 levels of subtree interchange, was used in the analyses involving just the UBA and UBL domains. Bootstrap tests were performed to establish the reliability of the final dendrograms obtained in the NJ, MP and ML analyses. A total of 1000 replicates were generated for NJ analyses and 100 replicates were made for the MP and ML analyses, which are more computer-intensive. MEGA 5.1 was also used to draw and edit the trees in Figures 1, 2, 3, 4 and 5.

The origin of the genes and the patterns of duplications and losses were determined by reconciling the gene trees with the species trees and, when needed, integrating additional information such as the relative location of the genes in several genomes, which ones are the genes located adjacent to those encoding ubiquilins or the ubiquilin protein structures (see Results for the details). Analyses of the genomic locations of ubiquilin genes were performed at the Ensembl genome browser web page [91] (http://www.ensembl.org/). Structural analyses of the ubiquilin proteins were performed using the integrated tool InterProScan [92] (available online at http://www.ebi.ac.uk/Tools/pfa/iprscan/).

Microarray data were obtained from the public repositories in which the datasets from the studies cited along the text were deposited. These were either the Gene Expression Omnibus (GEO) database at the NCBI (http://www.ncbi.nlm.nih.gov/geo/), the ArrayExpress database (http://www.ebi.ac.uk/arrayexpress/) or the BioGPS database (http://www.biogps.org). For some samples and genes, multiple probes or experiments were available. In those cases, I chose the ones with the highest average level of expression. The raw data of the experiments has been used in this study. Given that the quantitative values of expression of different experiments were not compared, no further normalization or other kinds of data manipulation were required.

Analyses to detect positive selection in human ubiquilins were performed using PAMLX [65], a graphical interface for the PAML program [63]. Methods were very similar to those described in one of my previous papers [64]. In brief, I took the protein sequences of the full-length UBA and UBL domains (a total of 117 amino acids) of the five ubiquilin genes present in both *Homo sapiens* and *Rattus norvegicus* (*UBQLN1, UBQLN2, UBQLN3, UBQLN4* and *UBQLNL*) and obtained the corresponding nucleotide sequences for those regions of the genes in the two species. Trees were obtained with the ten sequences that generated the expected topology (Figure 10). Then, the codon-substitution models implemented in the CODEML program of PAML [63] were used to estimate the synonymous (d_S_) and non-synonymous (d_N_) rates of evolution, in order to determine: 1) whether positive selection (ω = d_N_/d_S_ > 1) was detectable at some codons in the whole sets of sequences (“site models”); 2) whether positive selection was detected in particular branches of the sequences tree (“branch models”); and, 3) whether positive selection was detectable at some codons in particular branches of the trees (“branch-site models”) [63,64]. The CODEML analyses provide the log likelihood value of a given model of codon substitution, for the sequences considered and evaluating their evolutionary relationships. All the comparisons made here among alternative models corresponded to pairs of nested models. Thus, their results can be compared by using the LRT statistic. This statistic equals 2 Δl, being Δl the difference between the log likelihoods of the two models. The LRT statistic follows a chi-square distribution with a number of degrees of freedom equal to the difference in the number of parameters used in each of the two models that are compared (see details in [63,64] and references therein).

## Competing interests

The author declares that he has no competing interests.

## Supplementary Material

Additional file 1 Neighbor-joining tree in Newick format. Click here for file

Additional file 2 Ubiquilin alignment. Fasta file. Click here for file

Additional file 3 PAML results. Click here for file
